# Supplementary figures and images for: Sex chromosome dosage compensation in a sex reversing skink is not influenced by sexual phenotype
Source: BMC Genomics. 2025 Dec 17;27:72. doi: 10.1186/s12864-025-12217-1 (PMC12822173; doi:10.1186/s12864-025-12217-1)

Y-enriched kmer blast hits

Chromosome

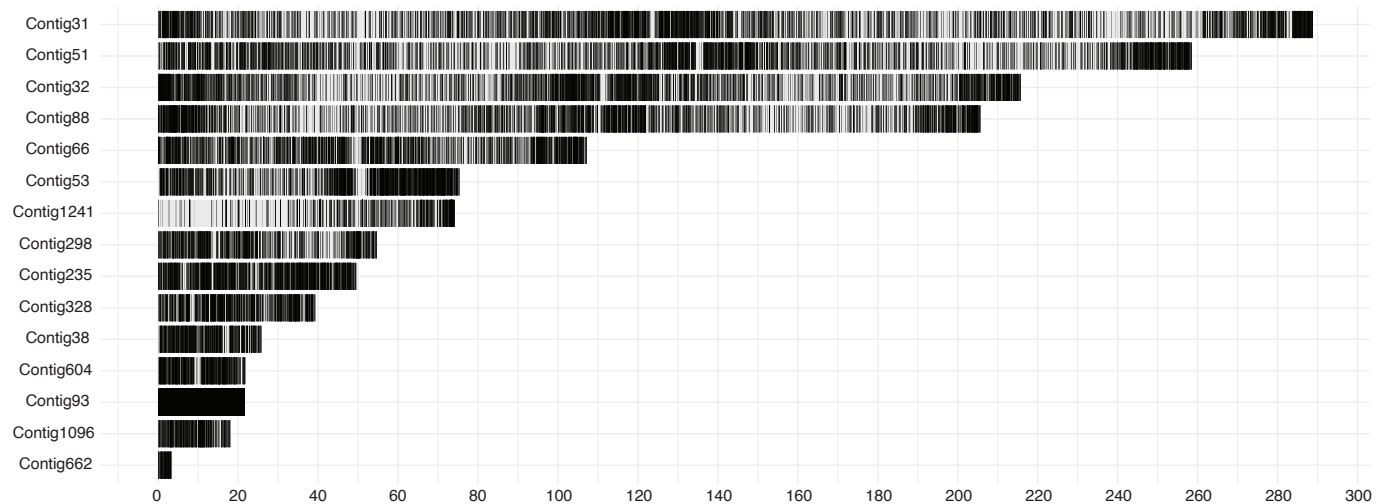

Genomic Position (Mbp)

Supplement: Supplementary file 1 — Supplementary Material 1: Supplemental Figure 1 Blastn hits to the the largest 15 scaffolds representing the B. duperreyi chromosomes with males specific kmer contigs. [file 12864_2025_12217_MOESM1_ESM.pdf]

Expression ratio of autosomal scaffolds

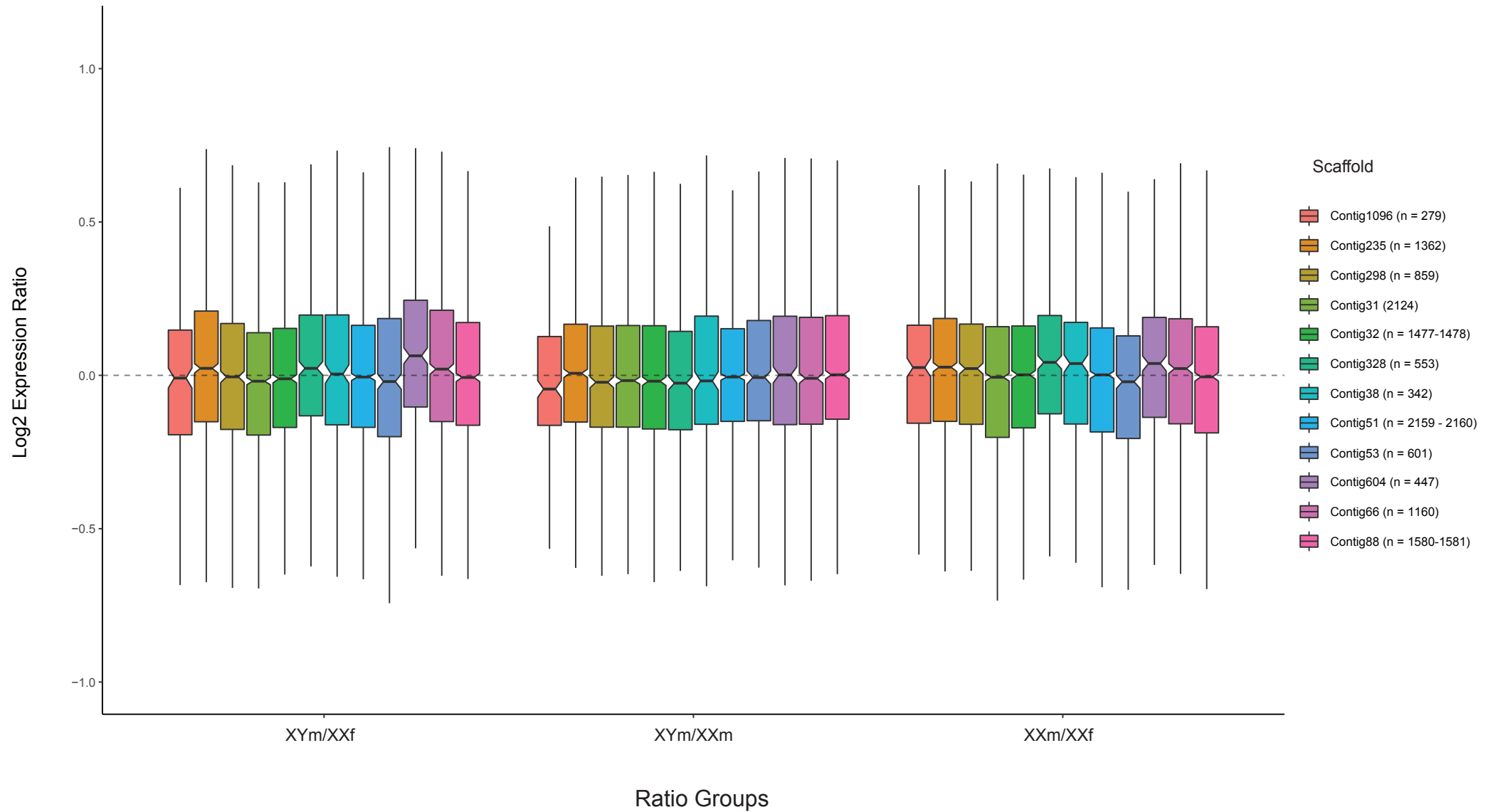

Supplement: Supplementary file 2 — Supplementary Material 2: Supplemental Figure 2. Expression ratios for each autosomal scaffold for the three pairwise sex/genotype comparisons presented as boxplots. Number of genes (n) is show in the key for each scaffold [file 12864_2025_12217_MOESM2_ESM.pdf]

**A** Autosomal Scaffold (contig 31)

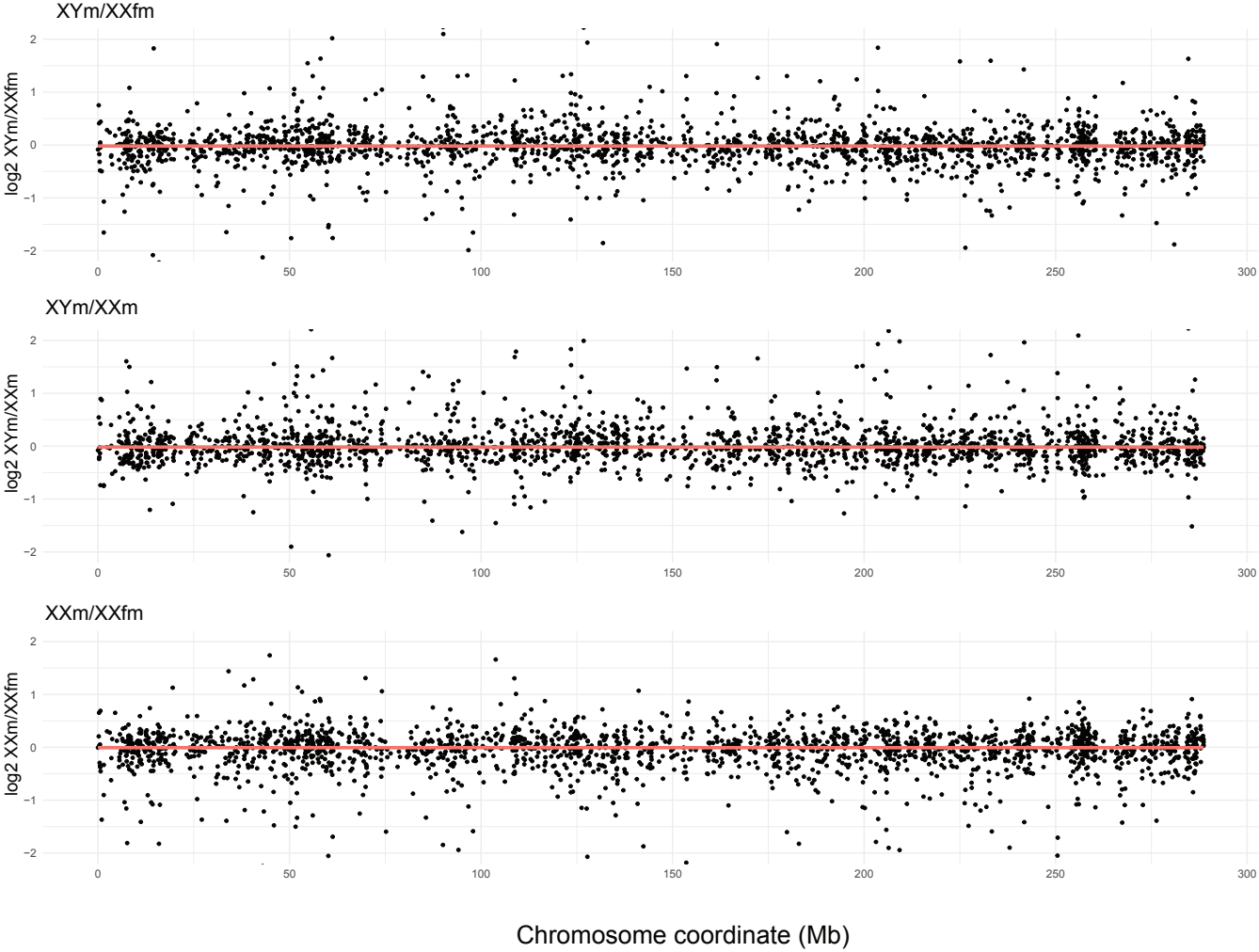

**B** X chromosome (contig 1241)

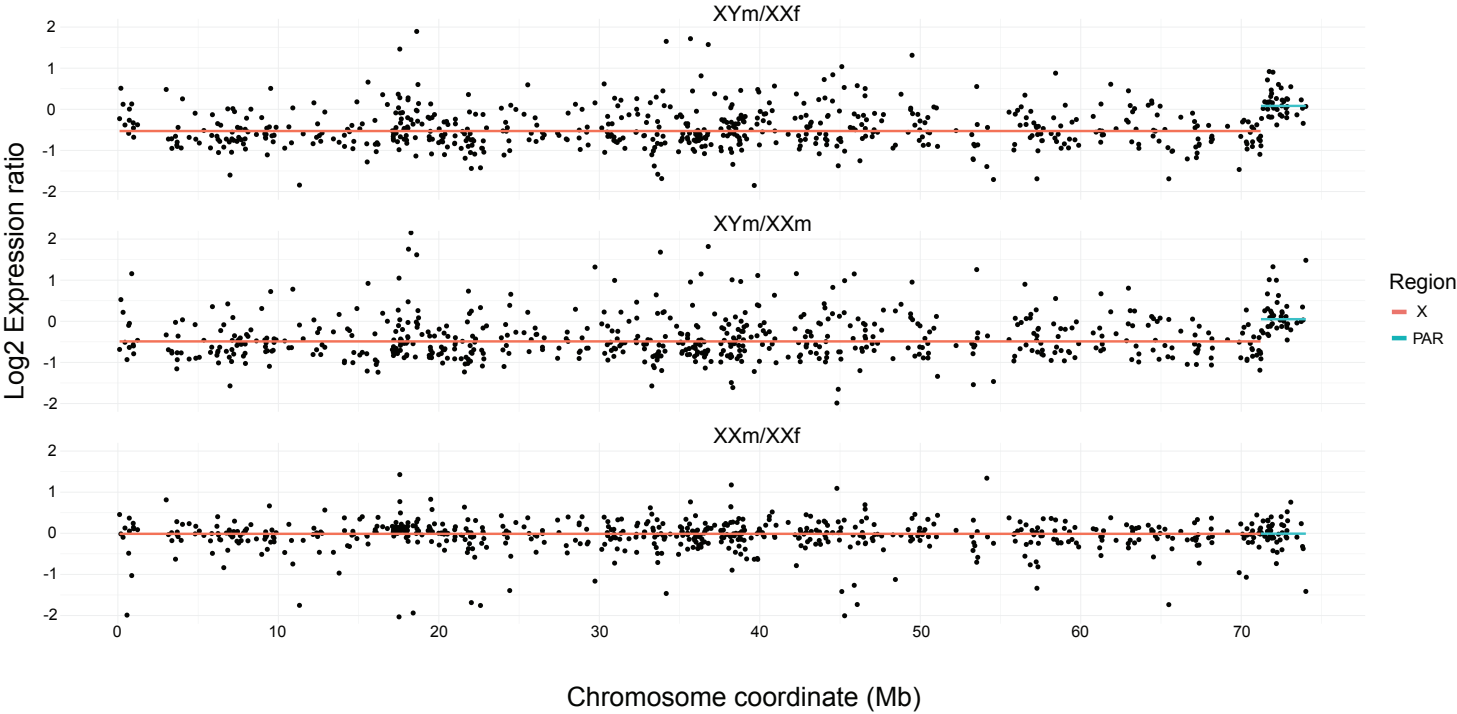

Supplement: Supplementary file 3 — Supplementary Material 3: Supplemental Figure 3. Expression ratios were calculated for each gene on A) a representative autosome (contig 31) and B) the X chromosome (contig 1241), in a pairwise fashion for each sex (XYm, XXm and XXf). Values are plotted on a log2 scale and median values for each comparison are plotted as a red line. For panel B median for the X specific region is a red line and PAR is a blue line [file 12864_2025_12217_MOESM3_ESM.pdf]
