# Supplementary material for: Sex chromosome dosage compensation in a sex reversing skink is not influenced by sexual phenotype
Source: BMC Genomics. 2025 Dec 17;27:72. doi: 10.1186/s12864-025-12217-1 (PMC12822173; doi:10.1186/s12864-025-12217-1)
Supplement: Supplementary file 4 — Supplementary Material 4: Supplemental Figure 4 Scatterplot of counts per million (CPM) for each gene for each pairwise comparison of XXf to XYm, XXm to XYm and XXf to XXm. X genes are plotted in black with slope plotted as a red line. Autosomal genes are plotted in blue with slope as a green line. Slope and R-squared of each trendline is shown above each plot [file 12864_2025_12217_MOESM4_ESM.pdf]

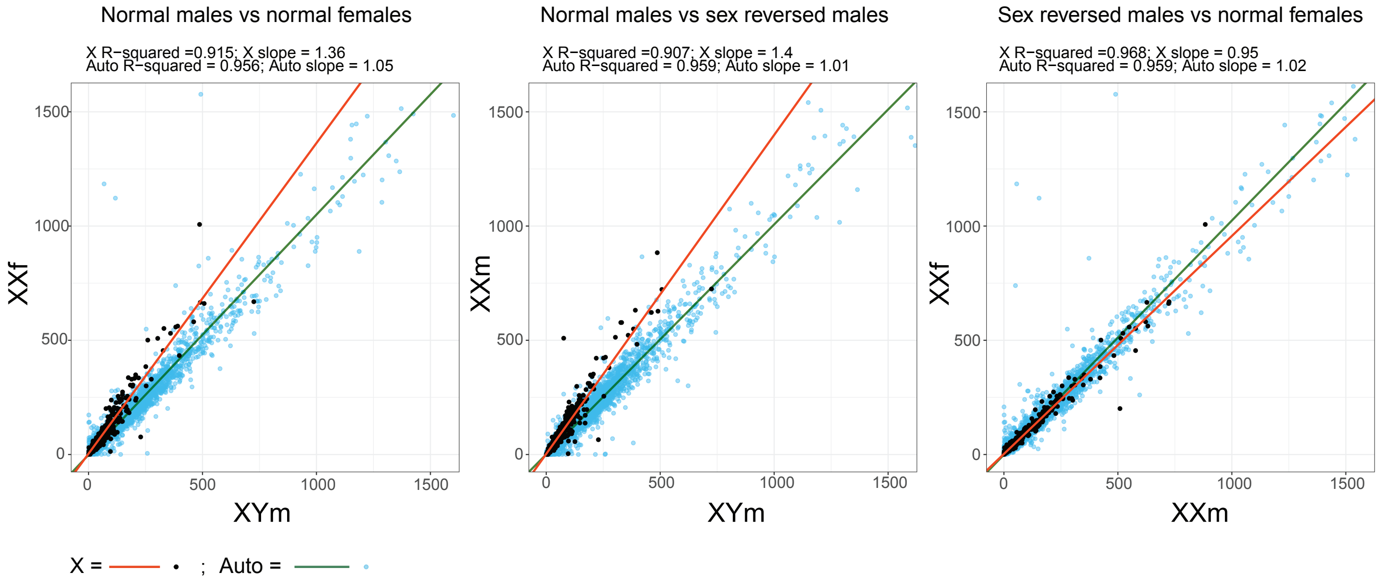
Normal males vs normal females
X R−squared =0.915; X slope = 1.36
Auto R−squared = 0.956; Auto slope = 1.05
XXf
XYm
Normal males vs sex reversed males
X R−squared =0.907; X slope = 1.4
Auto R−squared = 0.959; Auto slope = 1.01
XXm
XYm
Sex reversed males vs normal females
X R−squared =0.968; X slope = 0.95
Auto R−squared = 0.959; Auto slope = 1.02
XXf
XXm
X = ; Auto =
